# Isolation and identification of blueberry postharvest decay pathogen and control effect of 2,4-epibrassinolide

**DOI:** 10.3389/fpls.2025.1714776

**Published:** 2026-01-20

**Authors:** Shaofeng Jia, Zihuan Hou, Ke Li, Chunze Lu, Jing Ma, Lin Wu, Jinying Li, Yanan Li, Ying Wang

**Affiliations:** College of Horticulture, Jilin Agricultural University, Changchun, China

**Keywords:** blueberry, postharvest decay, 2,4-epibrassinolide (EBR), fungal disease, antioxidant enzyme

## Abstract

Due to its thin, juicy skin and high physiological activity, blueberries are easily susceptible to damage by pathogenic bacteria during storage and transportation after harvest. However, there are relatively few studies on the mechanisms by which blueberries resist rot disease. Therefore, in this study, the blueberry cultivar ‘Northland’ was used as the experimental material. Using a combination of single spore isolation, morphological analysis, and molecular biology methods, the types of pathogenic bacteria responsible for postharvest decay of blueberries were investigated, as well as the mechanisms by which EBR influences disease resistance and quality control. The results indicated that the pathogen responsible for blueberry fruit rot was *Talaromyces amestolkiae*, and treatment with 2,4-epibrassinolide (EBR) could significantly reduce both the fruit rot rate and the disease index. Among the treatments, 0.4 mg/L EBR showed the most pronounced effect, effectively maintaining fruit firmness, titratable acidity, and soluble solids content, while reducing weight loss, decreasing the extent of membrane lipid peroxidation, and enhancing the activities of antioxidant enzymes such as superoxide dismutase (SOD) and peroxidase (POD) by 251.46% and 208.18%, respectively. Additionally, the activity of pathogenesis-related proteins, including chitinase, increased, and the stability of antioxidants, such as total phenols, was preserved. EBR improved the disease resistance of blueberry fruit through multi-pathway synergy and delayed the deterioration of postharvest quality. The findings of this study can offer theoretical support for the eco-friendly prevention and management of postharvest diseases in blueberries.

## 1 Introduction

Blueberry (*Vaccinium* spp.*)* is a functional small berry rich in anthocyanins, polyphenols and vitamins (Li et al., 2025). It is known as the ‘ king of berries ‘, and its global consumer demand continues to grow. However, the skin of blueberries is thin and juicy, and during the ripening stage, they are often exposed to high temperatures and humidity, making them vulnerable to fungal diseases both before and after harvest. Post-harvest decay rates can reach as high as 20% to 30% (Bell et al., 2021), resulting in significant economic losses. Research indicates that the primary fungal diseases affecting blueberries include *Botrytis cinerea* (Yan and Hou, 2025), *Alternaria* spp (Li et al., 2025)., *Cladosporium* spp (Rivera et al., 2024)., and *Monilinia vaccinii-corymbosi* (Beg and Oliver, 2025). These pathogens can quickly spread during storage via latent infections or wound penetration, leading to fruit mildew, soft rot, and quality degradation (Wei et al., 2017). Studies have indicated that *Alternaria alternata* and *Botrytis cinerea* were isolated and identified from blueberry fruits in Liaoning Province, with the latent infection rate of host fruits reaching as high as 70%, which has become the primary cause of postharvest decay of blueberries in this region. Consequently, precise identification of pathogen species serves as the scientific foundation for developing targeted prevention and control strategies.

Currently, the prevention and control of blueberry diseases primarily rely on chemical fungicides and cultivation management practices. However, prolonged use of chemical agents can easily result in heightened pathogen resistance, excessive pesticide residues, and increased environmental risks. For example, *Botrytis cinerea* has developed significant resistance to benzimidazoles (Reyes-Ramírez et al., 2004). At the same time, consumer demand for environmentally friendly and safe fruits has driven research into biological control methods and plant immunity inducers. Although biological control shows potential (for example, the inhibition rate of the antagonistic yeast *Hanseniaspora uvarum* can reach 89%) (Yang et al., 2025), its stability in field applications is limited by environmental factors such as temperature and humidity. Induced systemic resistance (ISR), triggered by plant hormones, has emerged as a new area of research due to its high efficacy, low toxicity, and endogenous regulatory properties (Long et al., 2018).

Brassinosteroids (BRs) are a group of sterol phytohormones, among which 24-Epibrassinolide (EBR) has been the most extensively studied. It can activate the BRI1-BAK1 receptor complex to initiate downstream signaling pathways, regulate the activity of antioxidant enzymes (SOD, POD), modulate the activity of key enzymes in phenylpropanoid metabolism (PAL), influence defense gene expression, and enhance the synthesis of secondary metabolites (Liu et al., 2025). Recent studies have demonstrated that EBR can induce resistance to fungi, bacteria, and viruses in various plants. Treating tobacco can improve its resistance to tobacco mosaic virus (TMV), bacteria, and fungi, while treating rice can enhance its systemic resistance to fungi and bacteria (Liu et al., 2016; Gutierrez-Villamil et al., 2024). Additionally, EBR can increase the resistance of cucumber to *gray mold* and of pumpkin to *Phytophthora* (Najafi et al., 2024; Cao et al., 2025). However, research on EBR-induced resistance in blueberries against postharvest canker has not yet been reported.

In this experiment, ‘Northland’ blueberries (Vaccinium corymbosum L. ‘Northland’) were used as test materials to induce natural fruit diseases. The pathogens were purified using the single spore isolation method and identified through morphological observation and ITS-rDNA sequence analysis. The effects of different concentrations of 24-epibrassinolide (EBR) on fruit incidence and disease index, as well as its mechanism in regulating blueberry quality, were analyzed. This paper aims to elucidate the effects and physiological mechanisms of EBR on blueberry rot resistance and to provide theoretical support for the development of environmentally friendly postharvest rot prevention and control technologies for blueberries.

## 2 Materials and methods

### 2.1 Materials

The ‘Northland’ blueberry (Vaccinium corymbosum L. ‘Northland’) was collected from the Blueberry Science and Technology Courtyard in Jingyu County. Healthy fruits without mechanical damage and of uniform ripeness were selected and immediately stored at 0°C after harvest.

The medium used was potato dextrose agar (PDA): 200 g of peeled potatoes were cut and boiled for 30 minutes. The filtrate was strained, combined with 20 g of glucose and 15 g of agar, diluted to 1 liter with distilled water, and autoclaved at 121°C for 20 minutes. Major reagents included 24-epibrassinolide (EBR, purity ≥ 98%, Sigma), 75% ethanol, 1% sodium hypochlorite, Ezup column fungal genomic DNA extraction kit (Biotechnology), ITS universal primers (Biotechnology), and chitinase (CHT) and β-1,3-glucanase (GLU) activity assay kits (Solarbio). Key instruments comprised a texture analyzer (TA-XT plus), digital refractometer, UV-visible spectrophotometer (UV-2600), mold incubator, and high-speed refrigerated centrifuge (5810R).

### 2.2 Isolation and identification of pathogenic bacteria

#### 2.2.1 Isolation and purification of pathogenic bacteria

Reference tissue separation method (Harteveld et al., 2013): Ten naturally diseased ‘ Northland ‘ blueberry fruits were randomly selected. The diseased fruits were washed under running water for 5 minutes, then soaked in 75% ethanol for 1 minute → sterile water once → 1% sodium hypochlorite for 2 minutes → sterile water 3–5 times under an ultra-clean bench. To verify the disinfection effect, 100 μL of the final rinse water was applied to the PDA plate prepared according to section 2.1 and incubated at 28°C for 72 hours (sterile colony growth was considered satisfactory). Tissues (1 cm × 1 cm) were then plated onto PDA plates and cultured at 28°C for 72 hours. Single colonies with different morphologies were purified three times and stored at 4°C for future use. The separation and purification process was repeated three times.

#### 2.2.2 Morphological identification

The purified bacteria were inoculated onto three PDA plates (Preparation Reference 2.1) and cultured at 25°C for 7 days. Colony characteristics at different culture times (2, 4, and 7 days) were recorded by randomly selecting colonies. The mycelial area at the colony edge was randomly chosen during plate preparation. Colony color, diameter, and other characteristics were recorded daily. Mycelia measuring 5 mm × 5 mm were selected to prepare temporary slides, and microscopical observation was performed on the morphology and arrangement of mycelia, conidiophores, and conidia (Singh et al., 2021). Morphological observations were performed three times.

#### 2.2.3 Molecular biological identification

Three copies of genomic DNA were extracted from the purified strains for PCR amplification, with each PCR product subsequently sequenced for verification. A kit was used for genomic DNA extraction, and 1.0% agarose gel electrophoresis was employed to assess its purity and a micro-ultraviolet spectrophotometer (OD 260/OD 280 = 1.8–2.0). The ITS universal primers (ITS1: 5′-TCCGTAGGTGAACCTGCGG-3′; ITS4 for PCR amplification: 5′-TCCTCCGCTTATTGATATATGC-3′; For the PCR assay, a total volume of 25 μL was utilized, with 12.5 μL of 2× Taq PCR Master Mix incorporated, 1 μL of each primer, 2 μL of DNA template, and 8.5 μL of sterile ddH_2_O. The PCR protocol included an initial denaturation comprising an initial denaturation at 94°C for 5 minutes, then 35 cycles of denaturation at 94°C for 30 seconds, annealing at 55°C for 30 seconds, and extension at 72°C for 1 minute. Subsequently, a final extension was performed at 72°C for 10 minutes. Following the sequencing of PCR products, NCBI BLAST was used for alignment (homology ≥ 98%), ClustalX 2.1 was used for sequence alignment, and the neighbor-joining method in MEGA 11.0 was utilized to construct a phylogenetic tree (bootstrap analysis with 1000 repetitions) (Baldwin, 1992). The PCR amplification and sequencing process was repeated three times.

### 2.3 EBR treatment of blueberry fruit

In this study, the EBR treatment concentrations (0.1, 0.4, 0.8 mg/L) were established based on both literature research and previous preliminary test verification (dos Santos et al., 2021; Elena et al., 2021): The experiment was divided into four groups (three biological replicates, 100 grains per replicate): 0.1, 0.4, and 0.8 mg/L EBR treatment groups, and a sterile water control (CK). The EBR stock solution was dissolved in 95% ethanol and diluted to the target concentration (final ethanol concentration < 0.1%). The fruits were assigned to different treatment groups at random following a randomized block design. Within each group, fruits were randomly selected during the sampling process. After grouping, the fruits were immersed in the respective solution for 30 seconds and then dried at room temperature for 1 hour. After 6 hours, a pathogen spore solution of 1 × 10 cfu/mL (10 mL per 100 fruits) was inoculated (Yaping et al., 2022). Stored at 25°C with 95% relative humidity, at least 30 fruits were sampled on days 2, 4, 6, and 8, respectively, and a minimum of 30 fruits per replicate were collected for index determination. After rapid freezing in liquid nitrogen, they were stored at -40°C (Cai et al., 2019).

### 2.4 Determination of disease and quality index

The decomposition of 100 fruits per replicate was documented. The decay rate (%) is calculated as (number of decayed fruits ÷ total number of tested fruits) × 100 (Ippolito et al., 2000).

When grading the disease index, 50 fruits were randomly selected from each replicate for statistical analysis. Based on the percentage of the fruit surface affected by rot disease, five grades were established: grade 0: no disease; grade 1: 0.1-10.0%; grade 2: 10.1-25.0%; grade 3: 25.1-40.0%; grade 4: 40.1-60.0%; and grade 5: 60.1-100.0%: Disease severity index = [∑ (disease level × corresponding number of blueberry fruits at that level)]/(total number of blueberry fruits × highest disease level) × 100.

Fruit firmness was measured using a texture analyzer (P/5 probe, puncture depth of 5 mm). Soluble solids were measured with a digital refractometer using juice filtrate; Titratable acidity (TA) was measured via titration with 0.1 mol/L NaOH, with phenolphthalein acting as the indicator, and the outcomes were expressed as a percentage (calculated as citric acid). Weight loss rate (%) was calculated as [(weight before storage – sampling weight)/weight before storage] × 100. Hardness was measured on 10 grains per replicate, soluble solids and titratable acidity were measured on 5 grains per replicate, and weight loss rate was measured on 30 grains per replicate. Fruits in each group were randomly selected to ensure representativeness during index determination. There were 3 biological replicates (Harborne, 1970).

### 2.5 Assessment of oxidative stress, defense enzymes, and secondary metabolites

Oxidative stress and defense enzyme activity: Superoxide anion (O_2_^−^) was measured using the hydroxylamine oxidation method, and malondialdehyde (MDA) was measured using the thiobarbituric acid (TBA) method (Wang et al., 2025). Peroxidase (POD, guaiacol method), superoxide dismutase (SOD, NBT reduction method), phenylalanine ammonia lyase (PAL, boric acid buffer method), and catalase (CAT, spectrophotometry) were determined following the preparation of the crude enzyme extract. CHT and GLU activity reference kit instructions. For each treatment, 0.5–1 g of fruit tissue was collected at each time point to prepare the crude enzyme extract. Each sample was repeated three times. The samples were randomly selected and homogenized. The experiment was conducted three times.

Secondary metabolites: One gram of fruit tissue (including epidermis and pulp) was ultrasonically extracted with 80% methanol containing 0.1% HCl for 30 minutes. The supernatant was then centrifuged to determine total phenols (using the Folin-Ciocalteu method at 765 nm), flavonoids (using the NaNO2-Al(NO3)-Al method at 510 nm), and anthocyanins (calculated as OD_3_ - 0.25 × OD_3_ - 0.25) (Singleton et al., 1999). Three biological replicates were performed.

### 2.6 Statistical analysis

Statistical analysis was performed with SPSS version 26.0. One-way analysis of variance (ANOVA) was applied to determine differences among groups, while Duncan’s new multiple range test (p < 0.05) was adopted for multiple comparisons. Charts were generated with Origin 2022.

## 3 Results and analysis

### 3.1 Natural symptoms of blueberry fruit decay and verification of pathogenicity of pathogenic bacteria

**Figure 1:**
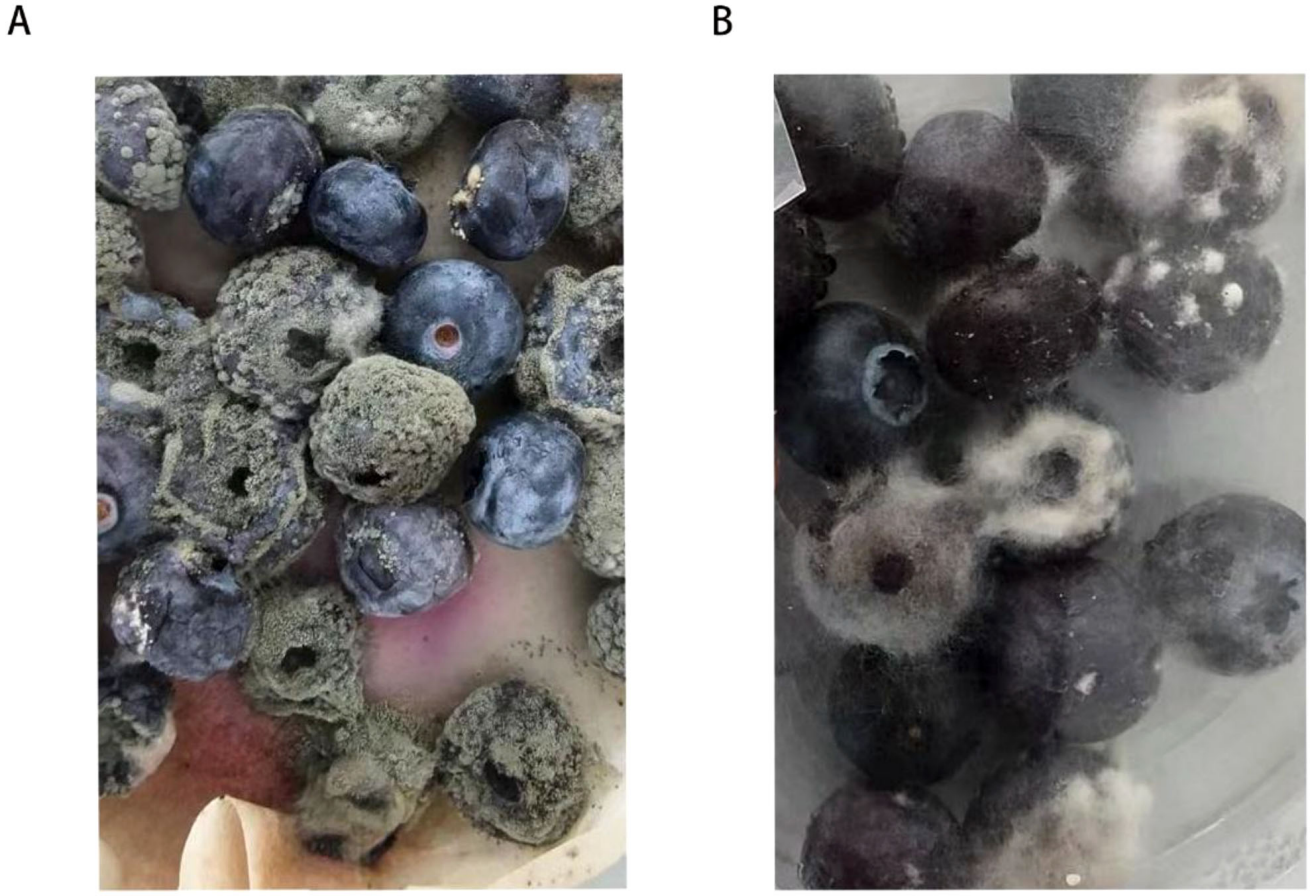
Blueberry fruit decay symptoms: **(A)** natural symptoms on the diseased fruit during storage; **(B)** After 7 days of inoculation with the H strain, symptoms appeared on sterile fruits.

A layer of black-gray mold and white, fluffy hyphae appeared on the surface of the diseased fruit (**Figure 1A**). Fungal strains H and Y were isolated from the diseased fruits using the tissue separation method. After 7 days of inoculation with H bacteria at 25°C, blueberry fruits exhibited symptoms of soft rot and the presence of white hyphae (**Figure 1B**, 7 days post-inoculation). These symptoms were identical to those observed in naturally infected fruits. Y bacteria did not exhibit pathogenicity. The diseased fruits were re-isolated, and the colony morphology and microscopic characteristics of the obtained strains matched those of the original H strain, in accordance with Koch’s postulates, confirming that the H strain is the pathogen responsible for the disease.

### 3.2 Morphological identification of the pathogen causing blueberry Valsa canker

**Figure 2:**
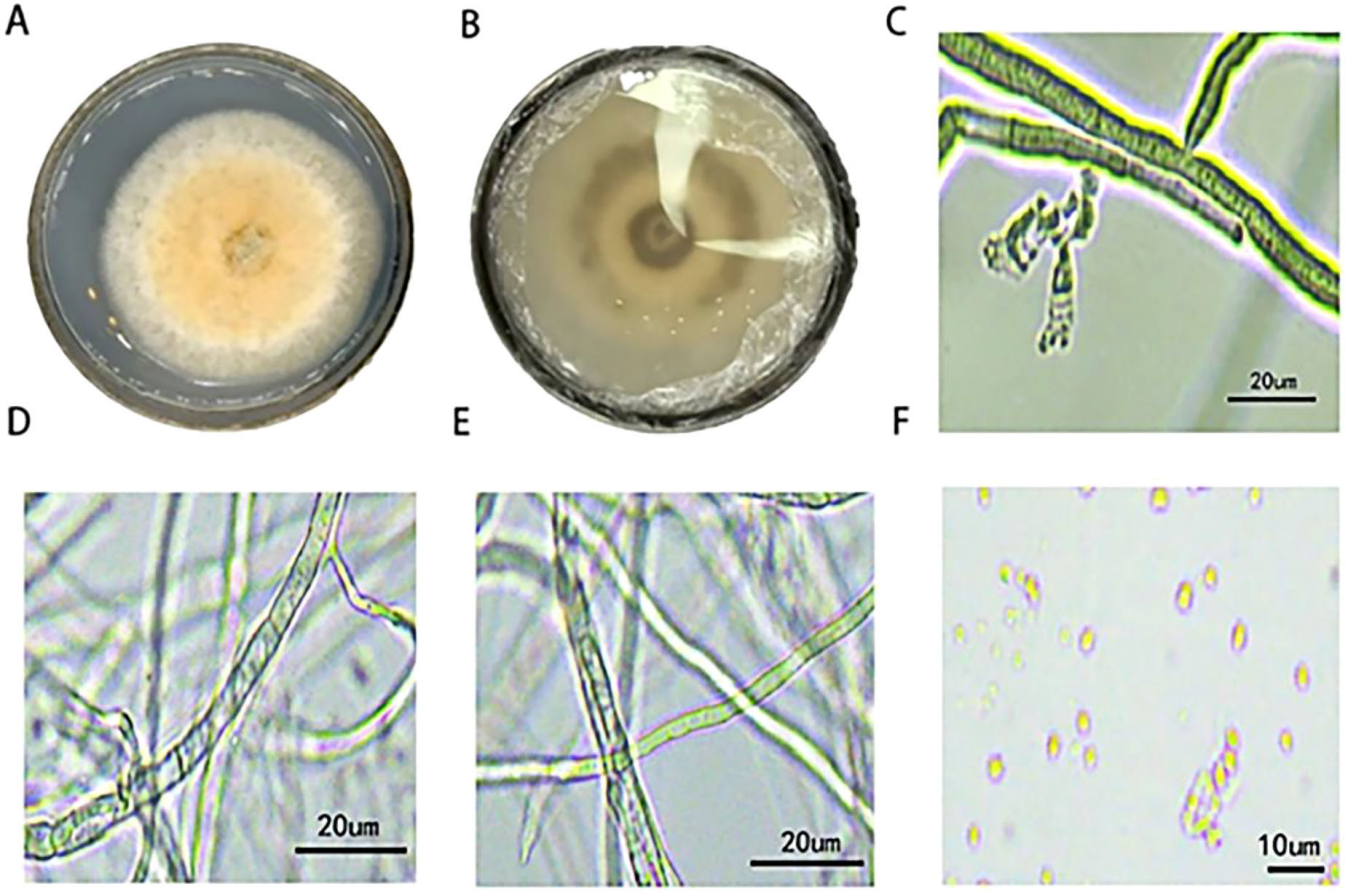
The morphological characteristics of strain H on PDA medium: the positive and negative views of 7-day-old colonies were **(A, B)**; **(C)** Conidiophores; **(D)** septate hyphae; **(E, F)** non-septate hyphae and conidia.

The colony’s surface was yellow-white, with dense aerial hyphae. Numerous powdery conidia were formed in the central area, along with faint ring patterns. Yellow pigment diffusion was observed on the reverse side of the colony, appearing light yellow (**Figures 2A, B**). Microscopic examination revealed that the conidiophores exhibited a tree-like branching pattern, with four or more conidia at the apex. The stalks were straight or slightly curved, measuring 22.50–32.50 × 8.50–12.50 μm (mean 26.80 × 10.50 μm, n = 15) (**Figure 2C**). The mycelium has a diaphragm (**Figure 2D**), while non-septate mycelial structures were also observed (**Figure 2E**); the length of the mycelial fragments ranges from 50.22 to 123.44 μm and a width of 4.11 to 5.21 μm. The conidia are oval, measuring 4.50–6.50 × 2.50–4.00 μm (mean 5.50 × 3.20 μm, n = 30) (**Figure 2F**).

### 3.3 Molecular biological identification of the blueberry Valsa canker pathogen

**Figure 3:**
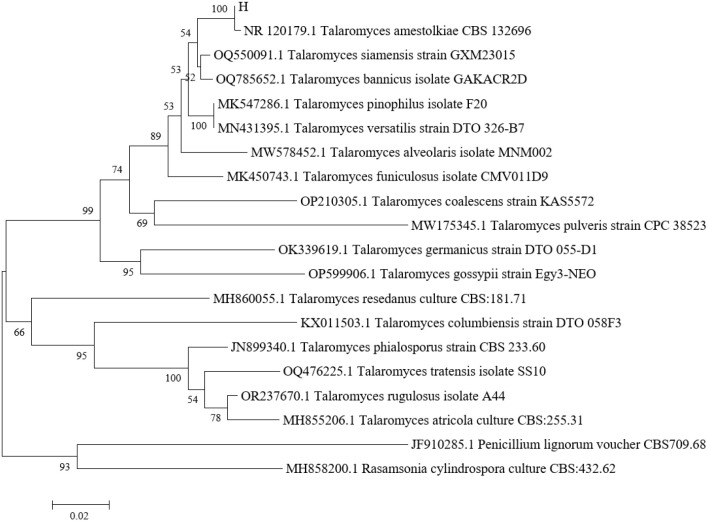
Utilizing the MEGA 11.0 software to construct the neighbor-joining phylogenetic tree of pathogenic fungi H.

Based on the sequencing results, a phylogenetic tree was constructed to determine the taxonomic status of strain H. *Aspergillus nidulans* and *Penicillium chrysogenum* were chosen as outgroups. Phylogenetic analysis indicated that strain H and *Talaromyces amestolkiae* CBS 132696 (NR_120179.1) grouped within the same clade with 100% bootstrap support (**Figure 3**). Based on its morphological features, including yellow-white colonies, tree-branch-producing conidiophores, and oval conidia, strain H was identified as *Talaromyces amestolkiae*.

### 3.4 Effects of EBR treatment on the decay rate and disease index of ‘Northland’ blueberry fruit during storage

**Figure 4:**
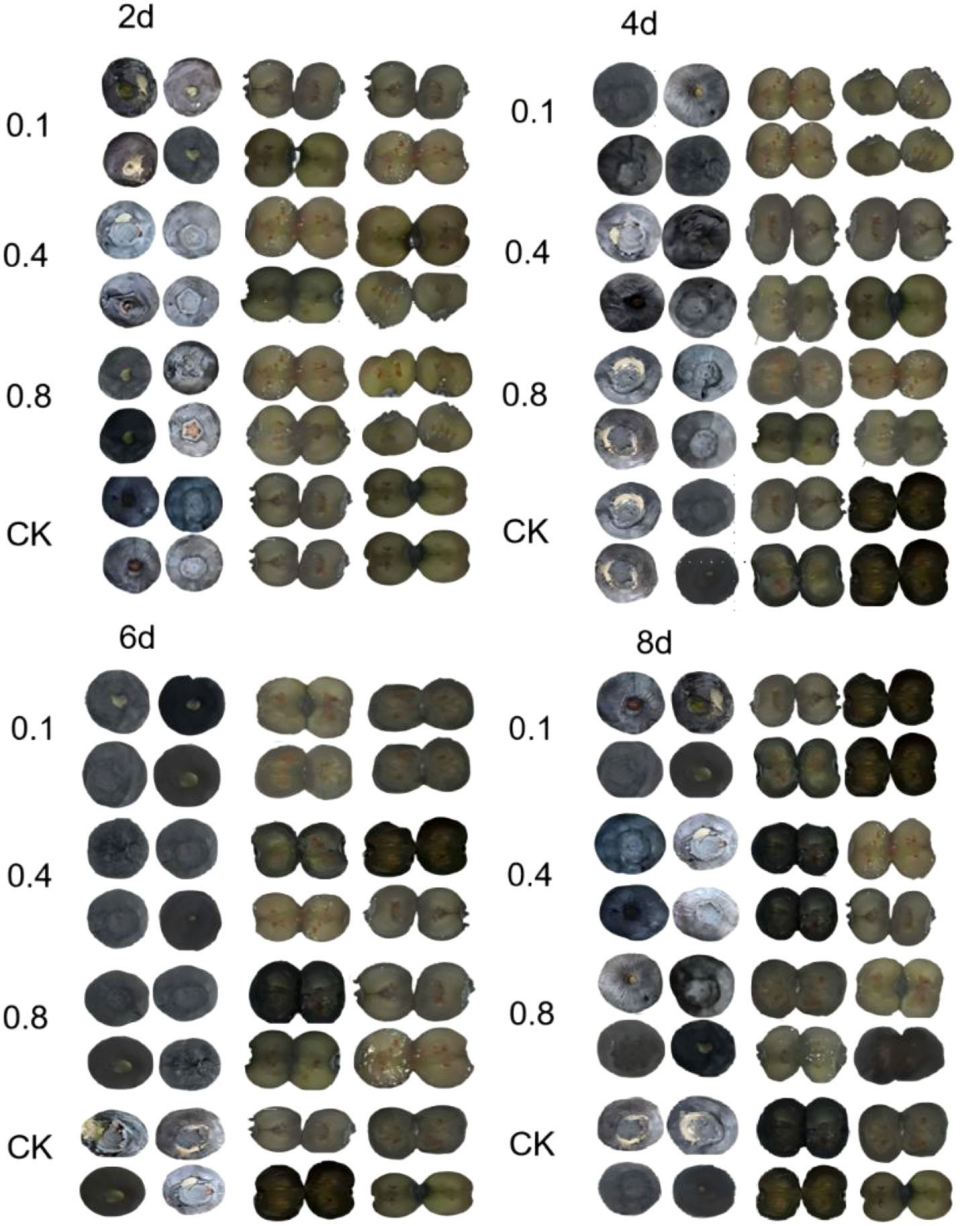
Shows that brassinolide application alleviated postharvest decay symptoms and reduced structural damage in blueberry fruits.

As illustrated in **Figure 4**, during storage, the fruits in the CK group exhibited evident signs of rotting and softening starting from the second day. On the fourth day, the fruits in the CK group and the 0.1 mg/L treatment group had generally decayed and softened, whereas the 0.4 mg/L and 0.8 mg/L EBR treatments significantly delayed disease progression. Among them, the 0.4 mg/L EBR treatment group exhibited the most significant effect, maintaining the smallest area of decay and the highest fruit firmness throughout the entire storage period, demonstrating the best appearance quality.

**Figure 5:**
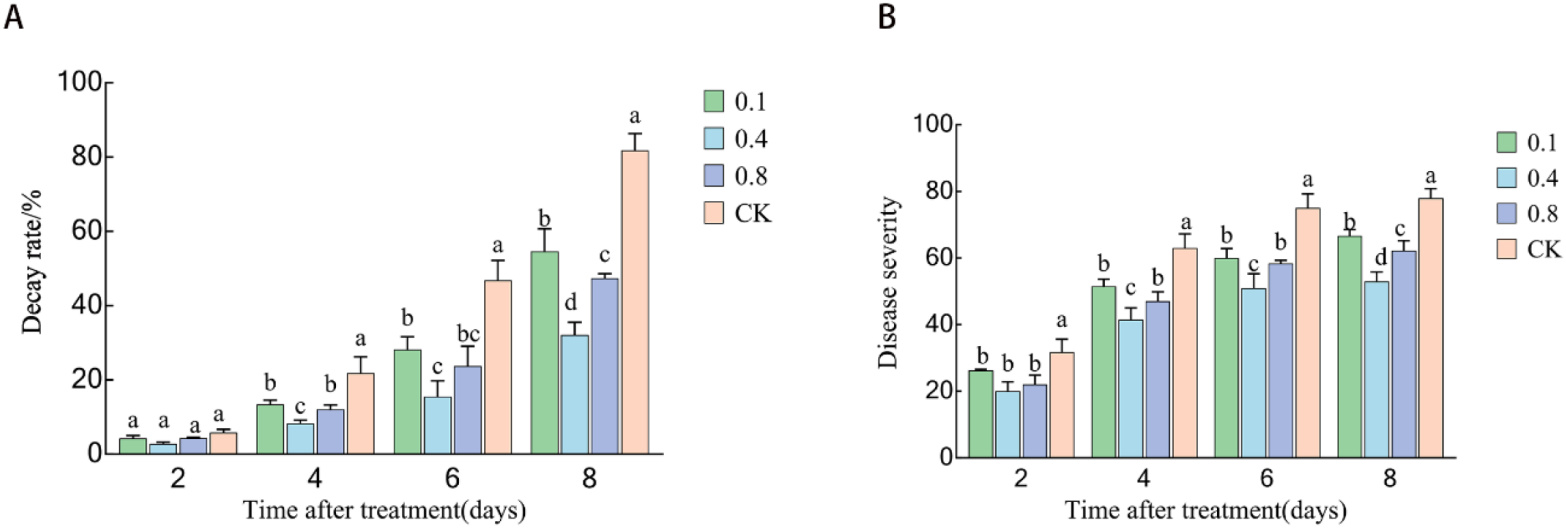
Effects of 24-epibrassinolide treatment on blueberry fruit: **(A)** Decay rate; **(B)** Disease index.​ Different lowercase letters indicate significant differences under different treatments (P < 0.05).

The decay rate of blueberry fruit gradually increased with extended storage time. As shown in **Figure 5A**, on the second day of storage, there was no significant difference in the decay rate between the treatment group and the control group (P > 0.05), indicating that the initial antibacterial effect of EBR had not yet manifested. On the fourth day of storage, the decay rate of the control group was significantly higher than that of each EBR treatment group (P < 0.05). On the 6th and 8th days of storage, significant differences were observed between the EBR treatment groups and the control group (P < 0.05). On the 8th day, the decay rates for the 0.4 mg/L and 0.8 mg/L treatment groups were 36.65% and 55% of that of the control group, respectively.

As illustrated in **Figure 5B**, the disease index of blueberry fruit gradually increased with the extension of storage time. On the 2nd, 4th, 6th, and 8th days, the disease indices of the 0.1, 0.4, and 0.8 mg/L EBR treatment groups were significantly lower than that of the control group (CK), with the 0.4 mg/L and 0.8 mg/L treatments showing superior efficacy. From the 4th to the 6th day, there was no significant difference between the 0.1 mg/L and 0.8 mg/L treatments, while the 0.4 mg/L treatment was significantly lower than the other groups. On the 8th day, significant differences were observed between treatments and control. Throughout the storage period, the disease index for the 0.4 mg/L treatment was the lowest, being 16.3% to 37.9% lower than that of the control (CK).

### 3.5 Effects of EBR treatment on the fruit quality of ‘Northland’ blueberries during storage

**Figure 6:**
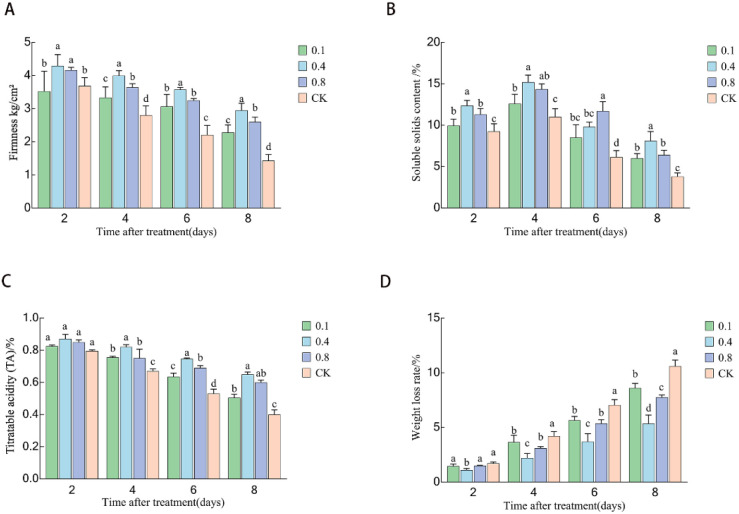
Effects of 24-epibrassinolide treatment on quality indices of blueberry fruit: **(A)** Hardness; **(B)** Soluble solids content; **(C)** Titratable acidity; **(D)** Weight loss rate. Different lowercase letters indicate significant differences between treatments (P < 0.05). Error bars represent ± SD (n=3).

As shown in **Figure 6A**, with the prolongation of storage time, the fruit hardness in each treatment group and the control group exhibited a declining trend. However, the hardness values in each EBR treatment group were consistently markedly higher than those in the control group (P < 0.05), indicating that EBR treatment effectively delayed fruit softening. In the preliminary stage of storage (days 2-4), not statistically different in hardness was observed between the EBR treatment groups (P > 0.05). During the middle and late stages of storage (days 6-8), the differences between the treatment groups became significant (P < 0.05), with the 0.4 mg/L treatment group maintaining the highest hardness. On days 6 and 8, the hardness of this group was 1.35 times and 1.72 times greater than that of the control group, respectively.

As illustrated in **Figure 6B**, the soluble solids content (SSC) of the fruit first rose and subsequently declined during storage. Throughout the entire storage duration, The SSC levels in all EBR treatment groups generally exceeded those in the control group. On the fourth day of storage, the SSC content reached its peak, with the 0.4 mg/L and 0.8 mg/L treatment groups exhibiting SSC levels 1.08 times and 1.06 times higher than the control group, respectively (P < 0.05). After that, the content gradually decreased. By the end of storage (day 8), there was no significant difference in SSC content between the 0.1 mg/L and 0.8 mg/L treatment groups (P > 0.05).

The weight loss rate of blueberries continued to increase as the storage time prolonged (**Figure 6C**). In the early stages of storage (2–4 days), there was no significant difference in weight loss rate between the 0.4 mg/L and 0.8 mg/L treatment groups (P > 0.05), and no significant difference was observed between the 0.1 mg/L treatment group and the control group (P > 0.05). At the later stage of storage (6–8 days), the weight loss rate of each EBR treatment group was significantly lower than that of the control group (P < 0.05), with the 0.4 mg/L treatment group showing the most pronounced effect. Compared with the control group, the weight loss rate was significantly reduced by 20% and 30% on the 6th and 8th days, respectively.

As shown in **Figure 6D**, the titratable acid (TA) content in the fruit continuously decreased with the extension of storage time. On the second day of storage, only the TA content in the 0.4 mg/L treatment group was significantly higher than that in the other groups (P < 0.05). From day 4 to day 8, the TA content in all EBR treatment groups was significantly higher than that in the control group (P < 0.05). At the end of the storage period (day 8), the TA content in the 0.4 mg/L and 0.8 mg/L treatment groups was 1.4 times and 1.1 times that of the control group, respectively, with a significant difference observed between the two treatment groups (P < 0.05).

### 3.6 Effect of EBR treatment on membrane lipid peroxidation in ‘Northland’ blueberry fruit during storage

**Figure 7:**
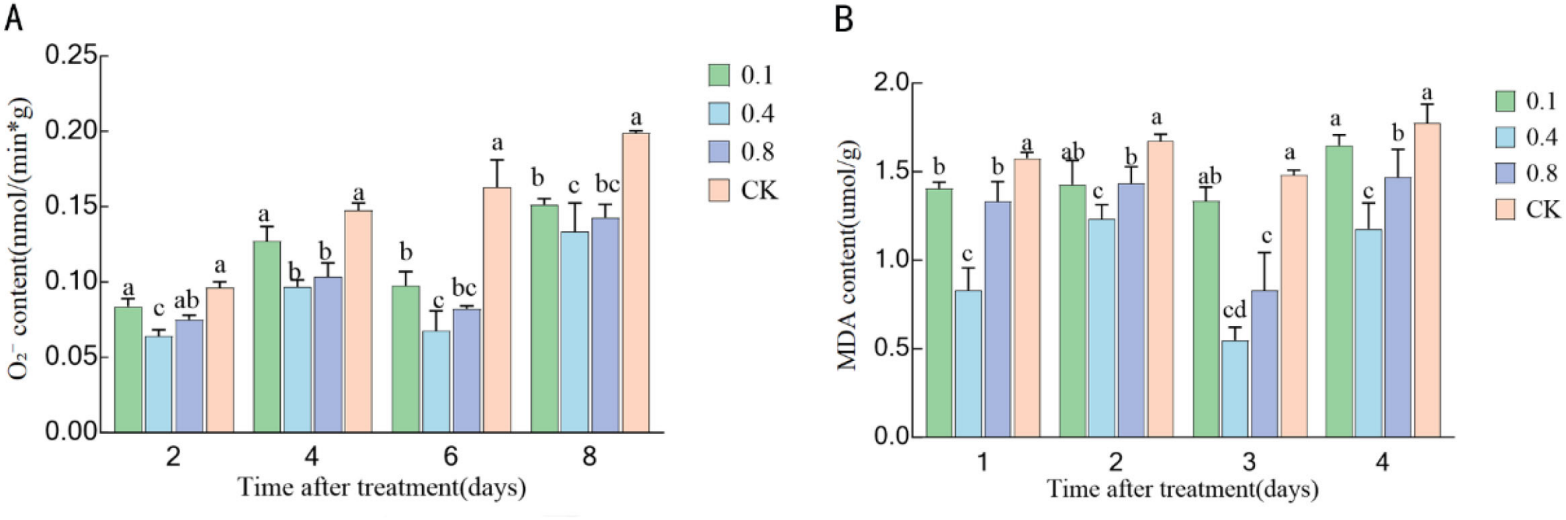
Effects of 24-epibrassinolide treatment on membrane lipid peroxidation in blueberry fruit: **(A)** Superoxide anion (O^_2_^−^^) production rate; **(B)** Malondialdehyde (MDA) content. Different lowercase letters indicate significant differences between treatments (P < 0.05). Error bars represent ± SD (n=3).

As illustrated in **Figure 7A**, the production rate of superoxide anion (O_2_^−^) varied over the course of storage. In the control group, the O_2_^−^ content initially increased, then decreased, and subsequently increased again. All EBR treatments were able to effectively inhibit the accumulation of O_2_^−^, with the 0.4 mg/L and 0.8 mg/L treatments showing the most significant effects. Throughout the entire storage period, the O_2_^−^ content remained at the lowest levels and was significantly lower than that of the control group and the 0.1 mg/L treatment group (P < 0.05). On the sixth day after treatment, the O_2_^−^ production rates in the 0.4 mg/L and 0.8 mg/L treatment groups reached their lowest levels, representing only 47.06% and 50% of the control group, respectively.

The trend in malondialdehyde (MDA) content was similar to that of O_2_^−^. As shown in **Figure 7B**, the MDA content in the control group initially increased, then decreased, and subsequently increased again. All EBR treatments inhibited MDA accumulation to varying degrees, with the MDA content in the 0.4 mg/L and 0.8 mg/L treatment groups being significantly lower than that in the control group and the 0.1 mg/L treatment group (P < 0.05). Similarly, on the sixth day, the MDA content in the 0.4 mg/L and 0.8 mg/L treatment groups reached their lowest levels, corresponding to 33.56% and 45.89% of the control group, respectively. This suggests that these two EBR treatment concentrations are most effective in reducing membrane lipid peroxidation damage in blueberry fruit. These findings indicate that EBR treatment significantly mitigates cell membrane peroxidation damage by efficiently scavenging superoxide anions, thereby helping to preserve the integrity of fruit cell structure.

### 3.7 Effects of EBR treatment on enzyme activities related to disease resistance in ‘Northland’ blueberry fruit during storage

**Figure 8:**
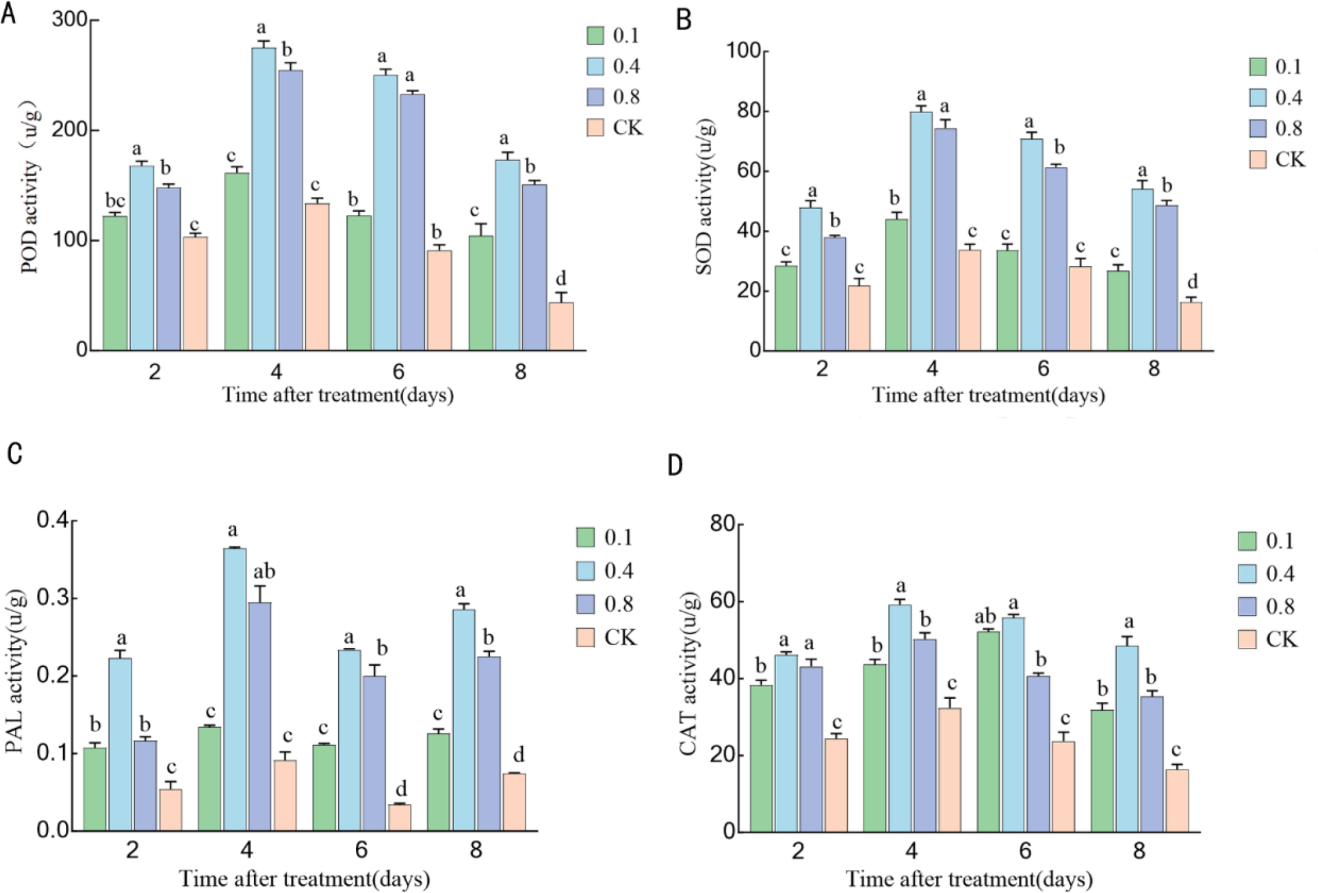
Effects of 24-epibrassinolide treatment on disease resistance-related enzyme activities in blueberry fruit: **(A)** Peroxidase (POD) activity; **(B)** Superoxide dismutase (SOD) activity; **(C)** Phenylalanine ammonia-lyase (PAL) activity; **(D)** Catalase (CAT) activity. Different lowercase letters indicate significant differences between treatments (P < 0.05). Error bars represent ± SD (n=3).

Peroxidase (POD) activity initially increased and then decreased throughout the entire storage period (**Figure 8A**). The POD activity in each EBR treatment group was consistently significantly higher than that of the control group (P < 0.05). The activity reached its peak on the fourth day of storage, with the 0.4 mg/L and 0.8 mg/L treatment groups exhibiting the highest levels, corresponding to 208.18% and 199.41% of the control group, respectively. Subsequently, the activity gradually declined. By the sixth day, there was no significant difference in POD activity between the 0.4 mg/L and 0.8 mg/L treatment groups (P > 0.05).

The change in superoxide dismutase (SOD) activity was similar to that of POD (**Figure 8B**), also exhibiting a trend of initially increasing and then decreasing. On the 4th day, which marked the peak, the SOD activity in the 0.4 mg/L and 0.8 mg/L treatment groups reached 251.46% and 235.58% of the control group, respectively, with no significant difference observed between the two treatment groups (P > 0.05). During the subsequent decline phase on the sixth day, there was no significant difference in SOD activity between the 0.1 mg/L treatment group and the control group (P > 0.05), indicating that the effect of the low-concentration treatment was diminished in the later stage of storage.

The activity of phenylalanine ammonia-lyase (PAL) initially increased, then decreased, and subsequently increased again (**Figure 8C**). Throughout the entire storage period, the PAL activity in all treatment groups was significantly higher than that in the control group (P < 0.05). On the fourth day, at the peak point, the effects of the 0.4 mg/L and 0.8 mg/L treatments were most pronounced, with their activities measuring 432% and 333% of the control group, respectively. Following this, the activity declined and then increased again after six days.

Catalase (CAT) activity initially increased and then decreased (**Figure 8D**). The CAT activity in each EBR treatment group was consistently significantly higher than that in the control group (P < 0.05). On the fourth day, the CAT activity of the 0.4 mg/L and 0.8 mg/L treatment groups reached their peak values, corresponding to 170.46% and 143.56% of the control group, respectively. Afterwards, the activity continued to decline. By the end of storage (day 8), there was no significant difference in CAT activity between the 0.1 mg/L and 0.8 mg/L treatment groups (P > 0.05). It should be noted that the activities of POD, SOD, CAT, and PAL peaked on the fourth day of storage. This coordinated response pattern indicates that EBR is likely to systematically activate the fruit’s inherent defense signaling pathway, rather than merely regulating the activity of a specific enzyme.

### 3.8 Effects of EBR treatment on chitinase and β-1,3-glucanase in ‘Northland’ blueberry fruit during storage

**Figure 9:**
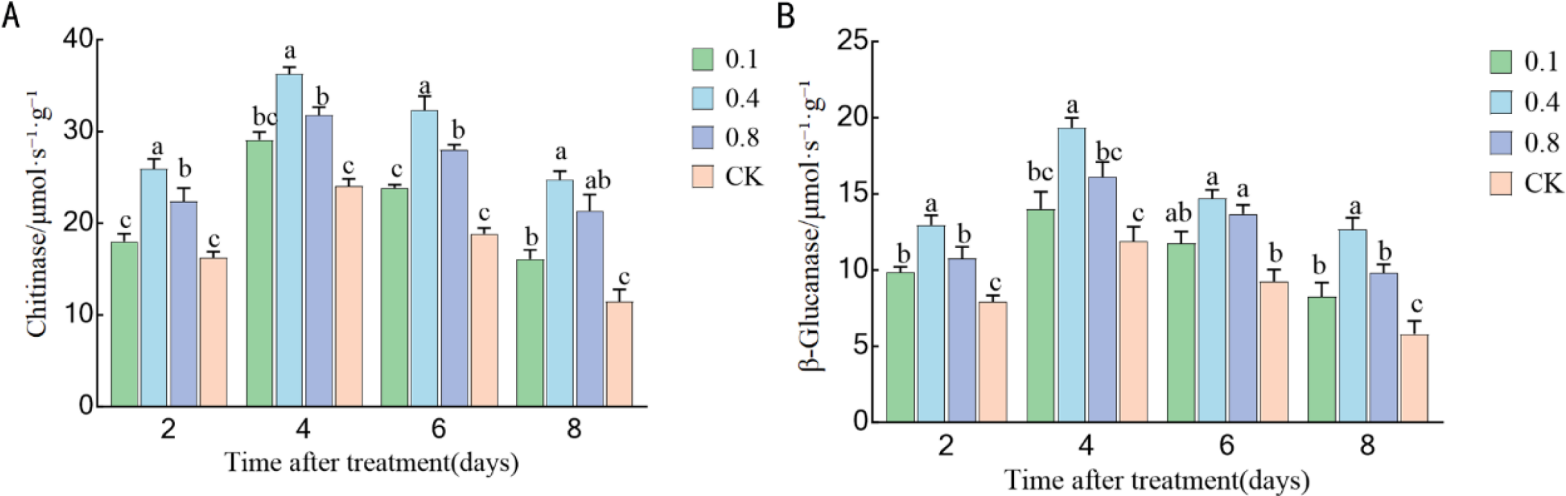
Effects of EBR treatment on defense-related enzyme activities in blueberry fruit against rot disease: **(A)** Chitinase activity; **(B)** β-1,3-glucanase activity. Different lowercase letters indicate significant differences between the control and EBR-treated groups at various storage time points (P< 0.05). Error bars represent ± SD (n=3).

As illustrated in **Figure 9A**, chitinase (CHT) activity initially increased and then declined during storage. The CHT activity in each EBR treatment group was significantly higher than that in the control group (P < 0.05). The activity peaked on the fourth day of storage, with the 0.4 mg/L and 0.8 mg/L treatment groups exhibiting the highest activity levels, reaching 180.44% and 133.45% of the control group, respectively. Subsequently, the activity gradually declined. By the end of the storage period (day 8), there was no significant difference in CHT activity between the 0.1 mg/L and 0.8 mg/L treatment groups (P > 0.05).

As illustrated in **Figure 9B**, the activity pattern of β-1,3-glucanase (GLU) was similar to that of CHT, exhibiting an initial increase followed by a decrease. The GLU activity in each EBR treatment group was also significantly higher than in the control group. On the fourth day after treatment, the GLU activity in the 0.4 mg/L and 0.8 mg/L treatment groups reached peak levels, corresponding to 150% and 137% of the control group, respectively. Subsequently, the activity began to decline. By the sixth day, no significant difference in GLU activity was observed between the 0.4 mg/L and 0.8 mg/L treatment groups (P > 0.05). The synergistic induction of chitinase and β-1,3-glucanase suggested that EBR treatment also enhanced the direct hydrolytic resistance of fruits against pathogens and, together with the antioxidant defense system, formed a more comprehensive disease resistance mechanism.

### 3.9 Effects of EBR treatment on secondary metabolites in ‘Northland’ blueberry fruit during storage

**Figure 10:**
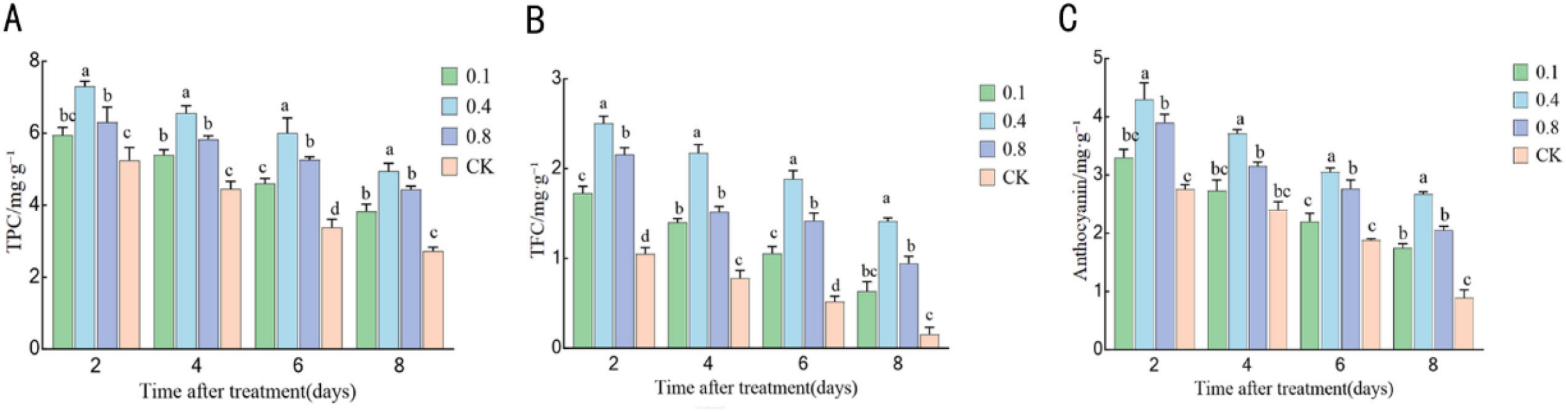
Effects of EBR treatment on secondary metabolites in blueberry fruit against rot disease: **(A)** Total phenol content; **(B)** Flavonoid content; **(C)** Anthocyanin content. Different lowercase letters indicate significant differences between the control and EBR-treated groups at various storage time points (P < 0.05). Error bars represent ± SD (n=3).

The total phenol content exhibited a declining trend throughout the storage period (**Figure 10A**). The total phenolic content in each EBR treatment group was significantly higher than that of the control group (P < 0.05). Among these, the 0.4 mg/L treatment group showed the most pronounced effect, maintaining the highest levels throughout the storage period. On the eighth day of storage, the total phenolic content in the 0.4 mg/L treatment group was 120% of that in the control group (P>0.05); however, the content in both treatment groups remained significantly higher than that of the control group.

The flavonoid content also exhibited a downward trend (**Figure 10B**). At most time points, the flavonoid content in each EBR treatment group was significantly higher than that of the control group. The 0.4 mg/L treatment consistently showed the most pronounced effect, maintaining the highest flavonoid content throughout the entire field. At the end of storage, the flavonoid content in the treatment group was 133% (day 6) and 150% (day 8) of that in the control group (P > 0.05); however, at all other time points, significant differences were observed between the treatment groups and the control group (P < 0.05).

The anthocyanin content exhibited a declining trend (**Figure 10C**). All EBR treatments significantly inhibited the reduction of anthocyanin, with their levels consistently remaining significantly higher than those of the control group (P < 0.05). The 0.4 mg/L treatment group demonstrated the most effective retention and the highest anthocyanin content. On the 6th and 8th days of storage, the anthocyanin content in the 0.4 mg/L treatment group was 177% and 200% of that in the control group, respectively. Similar to the change in total phenols, no significant difference was observed in anthocyanin content between the 0.1 mg/L and 0.8 mg/L treatment groups on the 4th and 8th days (P > 0.05). In summary, EBR treatment, particularly at a concentration of 0.4 mg/L, can effectively delay the degradation of key functional components in postharvest blueberries. The antioxidant activity of these secondary metabolites, as an important part of the enzymatic defense system, collectively helps maintain the postharvest quality and health of the fruit.

## 4 Discussion

Blueberry (*Vaccinium* spp.), being a nutrient-rich economic berry, is extensively cultivated worldwide for its distinctive flavor and health benefits. Nevertheless, in practical cultivation, its fruits are prone to pathogen infections and decay, leading to substantial economic losses throughout the industry chain (Scherm and Krewer, 2008; Gao et al., 2015). In this study, we identified *Talaromyces amestolkiae* as the primary pathogen responsible for blueberry fruit rot during storage in Jilin Province for the first time, using pathogen isolation, morphological observation, and molecular identification based on rDNA-ITS sequence analysis. Under conditions involving the tissue separation method combined with verification according to Koch’s postulates, no other pathogenic strains were detected. This result aligns with the findings of Zhou Qian (Qin et al., 2017) regarding the isolation and identification of pathogenic fungi on the surface of blueberry fruits stored at room temperature in Liaoning Province. Specifically, *Penicillium*-related fungi were identified as the primary pathogens responsible for postharvest decay of local blueberries, with the dominant pathogens further identified at the species level. This correlation confirms the predominance of *Penicillium* and related genera (such as *Basidiomycetes*) in post-harvest diseases of northern blueberries.

Postharvest decay of blueberries is a major factor limiting their ability to maintain quality and extend storage life. This process is closely associated with the fruit’s own senescence and the invasion of external pathogens. This mechanism is similar to the postharvest deterioration observed in most berries, such as strawberries and grapes (Ren et al., 2024; Yang et al., 2024; Cui et al., 2025). The results of this experiment indicated that treatment with 0.4 mg/L EBR could effectively regulate the decay rate and disease index of postharvest blueberries, while maintaining the stability of quality indicators such as soluble solids and titratable acidity. However, when the treatment concentration was increased to 0.8 mg/L, the regulatory effect was significantly reduced. The results were consistent with those reported by Zhang (Zhang et al., 2020). EBR was able to inhibit the increase in fruit decay rate during storage at room temperature (25°C), reduce the chilling injury index and incidence rate during low-temperature storage (0°C), and synergistically regulate the quality of fruit during low-temperature storage. Among the treatments, 0.9 mg/L showed the most effective results. In addition, 10 μmol/L EBR can also inhibit respiratory metabolism, thereby delaying the deterioration of eggplant quality and extending the storage period (Gao et al., 2015). These findings indicate that the regulatory effect of EBR on the postharvest quality of fruits may be influenced by the combined impact of treatment concentration and soaking duration. It is speculated that a high concentration of EBR may reduce its preservative effect by activating excessive stress-related metabolic pathways in blueberry fruit or disrupting the balance of endogenous hormones. This hypothesis requires further validation through a combination of transcriptomic analysis and hormone content measurement.

When plant tissues are infected by viruses, bacteria, fungi, or nematodes, reactive oxygen species (ROS) are produced, primarily including H_2_O_2_, -OH, and O_2_^−^ (Zhang et al., 2019). Research has demonstrated that brassinolide can decrease membrane lipid peroxidation by either inhibiting excessive ROS production or promoting the synthesis of free radical scavengers (Bi and Felton, 1995); specifically, 2,4-epibrassinolide can enhance CAT and SOD activity and gene expression, while reducing POD and PPO activity and gene expression, thus helping to preserve fruit storage quality (Li, 2017; Sun et al., 2021). In this experiment, EBR effectively preserved the structural integrity of the blueberry cell membrane by inhibiting the excessive accumulation of reactive oxygen species (O_2_^−^) and the formation of malondialdehyde (MDA), thereby establishing a foundation for the activation of subsequent defense mechanisms. Additionally, EBR’s regulation of the activities of antioxidant enzymes (SOD, POD, CAT) and defense enzymes (PAL, chitinase, glucanase) in blueberries exhibited clear concentration-dependent characteristics: the enzyme systems were synergistically activated at an appropriate concentration, with enzyme activity peaking at the 0.4 mg/L treatment, whereas a higher concentration (0.8 mg/L) of EBR resulted in a decline in enzyme activity which is consistent with the findings of Sun Yan (Sun, 2023) in sweet cherry. Xia et al. (Tang et al., 2022) found that EBR can reduce ROS accumulation in potatoes and slow down MDA accumulation by enhancing the activity of antioxidant enzymes. Chen Xurui et al. (Xu, 2022) confirmed that MeJA can enhance the activities of antioxidant and disease-resistance enzymes in cherries, thereby inhibiting infection by *Alternaria alternata*. From the perspective of the molecular mechanism hypothesis, the aforementioned regulatory effects of EBR may rely on the BRI1-BAK1 receptor complex-mediated signaling pathway: EBR binds to the BRI1-BAK1 complex on the cell membrane and triggers downstream signaling cascades. On one hand, it induces the transcriptional expression of genes involved in ROS scavenging, such as SOD and CAT, thereby maintaining ROS homeostasis and reducing membrane lipid peroxidation; on the other hand, it modulates the expression of defense-related enzyme genes (such as PAL and chitinase genes), which subsequently influences enzyme activity (Shi et al., 2025; Suresh et al., 2025). In this study, the regulatory effect of a high concentration of EBR (0.8 mg/L) was diminished. It is hypothesized that excessive EBR may result in saturation of the BRI1-BAK1 receptor or interfere with endogenous hormone signaling, thereby inhibiting the effective activation of downstream genes. This hypothesis requires verification through subsequent gene expression analysis.

Primary and secondary metabolism in plants are closely regulated in relation to each other. Secondary metabolites not only serve as essential substances for plant growth and development but also play a crucial role in helping plants adapt to their environment and resist both biotic and abiotic stresses (Dong et al., 2025). Li Lihua et al. (Zhang et al., 2022) demonstrated that 24-brassinolide (EBR) can stimulate the activity of key enzymes (such as phenylalanine ammonia-lyase and chalcone synthase) in the phenylpropanoid metabolic pathway, and promote the synthesis and accumulation of total phenols, flavonoids, and lignin during storage, thereby enhancing the postharvest disease resistance of apricot fruits. The results of this study demonstrated that exogenous EBR treatment can significantly enhance the accumulation of total phenols, flavonoids, and anthocyanins in blueberry fruits during storage, while effectively inhibiting the rise in fruit disease incidence and the expansion of lesion diameter following pathogen inoculation. The results are highly consistent with the findings of Zhang et al. (Liu et al., 2025) regarding the regulation of phenolic synthesis and disease resistance in Rosaceae fruits such as strawberries and peaches by EBR, further confirming that EBR’s mechanism for enhancing postharvest disease resistance in fruits through the regulation of secondary metabolism is broadly applicable. From the perspective of the mechanism of action, phenols can enhance the host’s disease resistance in two ways. First, they can directly inhibit spore germination, mycelial growth, and polygalacturonase activity of pathogens, thereby reducing the pathogens’ infectivity. Second, they can participate in the host’s defense response by promoting wound callus formation, as well as cell wall lignification and corkification, thereby forming a physical defense barrier that prevents the spread of pathogens (Zhao et al., 2007). Based on the observed effect of EBR in promoting the accumulation of phenolic compounds in blueberry fruits in this study, it can be inferred that EBR may stimulate the synthesis of disease-resistant secondary metabolites by activating secondary metabolic pathways, such as phenylpropanoid metabolism, in blueberries. Ultimately, this may facilitate the prevention and control of postharvest diseases, providing a crucial experimental foundation for analyzing the molecular mechanisms through which EBR regulates postharvest disease resistance in blueberries.

## 5 Conclusions

In this study, *Talaromyces amestolkiae* was identified for the first time as the primary spoilage microorganism affecting ‘Northland’ blueberries in Jilin Province, it was also confirmed that 2,4-epibrassinolide (EBR) exerted a concentration-dependent regulatory effect on the postharvest preservation of blueberries. An optimal concentration is 0.4 mg/L - On the 8th day of storage, the decay rate was only 36.65% of that of the control group, the disease index was reduced by 16.3% to 37.9%, and the hardness was 1.72 times that of the control group. The production rate of superoxide anion (O_2_^−^) and the content of malondialdehyde (MDA) were 47.06% and 33.56% of the control group, respectively. The activities of SOD and CHT, as well as the anthocyanin content, reached their highest levels, which were 251.46%, 180.44%, and 200% of the control group, respectively. However, the effect of 0.8 mg/L EBR decreased significantly. For instance, the decay rate increased to 55% of the control group, and enzyme activity decreased by 15%–20% compared with the 0.4 mg/L group. In this study, it was evident that *Talaromyces amestolkiae* was the predominant spoilage microorganism, addressing a gap in postharvest pathogen research for blueberries in the northeastern cold region and providing a foundation for targeted disease prevention and control. Additionally, the preservation effects and optimal concentration of EBR were determined, offering theoretical support for the research and development of postharvest green preservation technologies for blueberries.

However, the mechanism behind the reduced effect of high concentrations of EBR (such as 0.8 mg/L) remains unclear in this study. Moving forward, attention should be given to investigating the interference with the synthesis and signal transduction of abscisic acid and ethylene.

## Data Availability

The datasets presented in this study can be found in online repositories. The names of the repository/repositories and accession number(s) can be found in the article/**Supplementary Material**.
