# Supplementary material for: Isolation and identification of blueberry postharvest decay pathogen and control effect of 2,4-epibrassinolide
Source: Front Plant Sci. 2026 Jan 20;16:1714776. doi: 10.3389/fpls.2025.1714776 (PMC12864428; doi:10.3389/fpls.2025.1714776)
Supplement: Supplementary file 1 [file Table1.docx]

**Additional materials**

2.3 **EBR treatment of blueberry fruit**

Five concentration gradients of 0.05, 0.1, 0.4, 0.8, and 1.2 mg/L were prepared to determine the fruit decay rate and the activities of SOD and POD after four days of storage**.** The results indicated that there was no significant difference between the 0.05 mg/L treatment and the control group (CK) (P > 0.05). Enzyme activity was inhibited by the 1.2 mg/L treatment, and the regulatory effect of EBR on blueberry disease resistance-related indices was significant, showing a gradient difference within the range of 0.1 to 0.8 mg/L. Therefore, three representative concentrations within this range (low concentration 0.1 mg/L, medium concentration 0.4 mg/L, high concentration 0.8 mg/L) were selected to conduct formal experiments in order to determine the optimal concentration and the concentration-dependent mechanism by which EBR regulates postharvest disease resistance in blueberries.
